# Detection of temozolomide-induced hypermutation and response to PD-1 checkpoint inhibitor in recurrent glioblastoma

**DOI:** 10.1093/noajnl/vdac076

**Published:** 2022-05-23

**Authors:** Paul Daniel, Brian Meehan, Siham Sabri, Fatemeh Jamali, Jann N Sarkaria, Dongsic Choi, Delphine Garnier, Gaspar Kitange, Kate I Glennon, Antoine Paccard, Jason Karamchandani, Yasser Riazalhosseini, Janusz Rak, Bassam Abdulkarim

**Affiliations:** Department of Oncology, McGill University, Research Institute of the McGill University Health Centre (Research Institute-MUHC), Montreal, Canada; McGill University, Research Institute of the McGill University Health Centre (Research Institute-MUHC), Montreal, Canada; Research Institute, Cancer Research Program, McGill University Health Centre, Glen Site, 1001 Decarie Boulevard, Montreal, Quebec H4A 3J1, Canada; McGill University, Research Institute of the McGill University Health Centre (Research Institute-MUHC), Montreal, Canada; Mayo Clinic, Department of Radiation Oncology; McGill University, Research Institute of the McGill University Health Centre (Research Institute-MUHC), Montreal, Canada; McGill University, Research Institute of the McGill University Health Centre (Research Institute-MUHC), Montreal, Canada; Mayo Clinic, Department of Radiation Oncology; McGill University, McGill Genome Centre; McGill University, McGill Genome Centre; Department of Pathology, McGill University, Montreal, Canada; McGill University, McGill Genome Centre; Department of Pediatrics, McGill University Health Centre, Glen Site, 1001 Decarie Boulevard, Montreal, QC H4A 3J1, Canada; Department of Oncology, McGill University, Research Institute of the McGill University Health Centre (Research Institute-MUHC), Montreal, Canada

**Keywords:** glioblastoma, hypermutation, immune therapy, recurrence, Temozolomide

## Abstract

**Background:**

Despite aggressive upfront treatment in glioblastoma (GBM), recurrence remains inevitable for most patients. Accumulating evidence has identified hypermutation induced by temozolomide (TMZ) as an emerging subtype of recurrent GBM. However, its biological and therapeutic significance has yet to be described.

**Methods:**

We combined GBM patient and derive GBM stem cells (GSCs) from tumors following TMZ to explore response of hypermutant and non-hypermutant emergent phenotypes and explore the immune relevance of hypermutant and non-hypermutant states in vivo.

**Results:**

Hypermutation emerges as one of two possible mutational subtypes following TMZ treatment in vivo and demonstrates distinct phenotypic features compared to non-hypermutant recurrent GBM. Hypermutant tumors elicited robust immune rejection in subcutaneous contexts which was accompanied by increased immune cell infiltration. In contrast, immune rejection of hypermutant tumors were stunted in orthotopic settings where we observe limited immune infiltration. Use of anti-PD-1 immunotherapy showed that immunosuppression in orthotopic contexts was independent from the PD-1/PD-L1 axis. Finally, we demonstrate that mutational burden can be estimated from DNA contained in extracellular vesicles (EVs).

**Conclusion:**

Hypermutation post-TMZ are phenotypically distinct from non-hypermutant GBM and requires personalization for appropriate treatment. The brain microenvironment may be immunosuppressive and exploration of the mechanisms behind this may be key to improving immunotherapy response in this subtype of recurrent GBM.

**Key Points**

1. Hypermutated recurrent GBM has greater immunogenicity than non-hypermutant tumors.
2. The brain micro-environment suppresses immune response against hypermutated GBM.
3. DNA from hypermutated recurrent GBM is expelled in extracellular vesicles and reflects the genomic landscape of originating cells.

**Importance of the Study**

We report several unexplored aspects regarding the therapeutic relevance of hypermutation in recurrent GBM. First, we confirm using multiple experimental models of glioma that TMZ treatment leads to two mutational subtypes at recurrence; non-hypermutant and hypermutant. These recurrent subtypes are phenotypically distinct and response differently to several drugs suggesting the need for distinct treatment approaches. Second, we find that the orthotopic brain microenvironment contributes to immune suppression, resulting in loss of immunogenicity even in hypermutant GBM tumors, which otherwise have the capacity to evoke immune response in subcutaneous contexts. Finally, we show that acellular vesicles released from hypermutant recurrent GBM contain genomic material which can be used to detect and diagnose hypermutation, opening the opportunity for further development towards liquid biopsy diagnostic tests.

Glioblastoma (GBM) is the most common and deadly type of malignant brain cancer in adults. Despite aggressive upfront treatment which combines resection, radiation therapy (RT) and Temozolomide (TMZ), recurrence is inevitable and ultimately lethal.^1^ Patient survival has stagnated since the implementation of Stupp protocol in 2005 and with only limited improvement reported in a recent trial combining RT, TMZ and lomustine at the cost of excessive toxicity.^2^ With no approved therapy able to substantially prolong patient survival at recurrence, unraveling efficient therapeutic opportunities for recurrent glioma is a priority.

Recent studies comparing genetic features of primary and recurrent GBM following treatment with TMZ identified multiple outcomes at recurrence, including *hypermutation*. This hypermutant state is characterized by mutational inactivation causing loss of mismatch repair (MMR) gene function and accumulation of single nucleotide variations (SNVs) throughout the genome.^3–7^ In comparison, *non-hypermutant* recurrent tumors are characterized by maintenance of MMR pathway functionality and minimal accumulation of mutational burden. Notably, whilst high tumor mutational burden (TMB) is a known predictor for immune checkpoint inhibitor (ICI) response in several cancer types, there is only limited evidence from clinical case reports suggesting the capacity for hypermutation to predict response to ICI in GBM. More studies are needed to evaluate whether hypermutant recurrent GBM represent therapeutically relevant subtypes, which can inform subsequent treatment approach.

Currently, identification of a hypermutant recurrent GBM relies on whole exome (WES) or whole genome sequencing (WGS), limiting large-scale implementation in most clinical settings. Whilst targeted sequencing approaches which interrogate specific regions of the genome are currently available (eg Foundation 1, T200, MSK-IMPACT), they remain largely focused upon oncogenic and tumor-suppressor regions and not specifically designed to detect hypermutation in GBM. The spectrum of mutagenesis in TMZ-driven hypermutation has not been observed to be concentrated in these regions and use of these generic cancer gene panels may potentially bias results. Additionally, when these panels have been used, cutoffs for defining hypermutation are often arbitrarily chosen (eg >30 mutations using Foundation 1 panels^8^), in part due to poorly defined criteria to define hypermutation in recurrent GBM. Similarly, microsatellite instability (MSI) testing has limited utility to detect hypermutation in GBM.^5^ Establishing a low-cost targeted gene panel specifically designed to detect hypermutation in GBM would accelerate the introduction of hypermutation as a potentially clinically actionable biomarker.^9^

We previously demonstrated that GBM stem cell (GSC) xenografts subjected to treatment with TMZ undergo divergent evolutionary phenotypic changes including hypermutant TMZ resistance patterns.^10^ These phenotypic changes were reflected in the transcriptome of extracellular vesicles (EV) released from these cells.^10^

In this study, we utilize immunocompromised and immunocompetent models of hypermutant and non-hypermutant glioma and characterize their mutational, phenotypic and therapeutic profiles. Using an immunocompetent in vivo system, we demonstrate that hypermutant GSC injected subcutaneously induce immune response and display a high rate of immune rejection which is not observed in non-hypermutant GSCs. We also establish the importance of the brain microenvironment in establishing failed immune response as hypermutant GSCs fail to induce immune response in orthotopic contexts which was unable to be rescued by ICI treatment. Finally, we report a limited gene signature, which identifies hypermutation and demonstrate that this signature is detectable in EVs, paving the way to non-invasive liquid biopsy-based approaches to detect these genes.

## Methods

### Cell Culture and Treatment

GSCs were cultured in Dulbecco’s modified Eagle’s medium/F12 supplemented with B27 (2%), Glutamax, epidermal growth factor (20 ng/mL), basic fibroblast growth factor (20 ng/mL), penicillin/streptomycin (Life Technologies) and heparin 5 μg/mL (Stem Cell Technologies) following our institutional guidelines (RI-MUHC Biohazard Safety Certificate). Murine GL261 cells were cultured using DMEM high glucose medium supplemented with FBS (10%). Cell lines were established and provided by Ichiro Nakano. Manipulations and cell line derivatives are listed in the Supplementary Table 1.

In vitro radiation studies were performed using a Faxitron irradiator and clonogenic assessment of survival performed after 14–21 days. In vitro drug treatment studies were performed by assessment of viability 4 days after treatment using standard Resazurin assay conditions.

### Molecular Analysis

Methylation specific PCR was performed as previously described.^11^ Briefly, purified DNA was bisulfite converted (EZ DNA methylation-Lightning Kit, Zymo) followed by PCR using the following conditions (95˚C for 15 min, then 35 cycles of 95˚C for 50 s, 59˚C for 50 s and 72˚C for 50 s, followed by a final step at 72˚C for 10 min).

Immunoblot analysis of protein lysates was performed as previously described.^10^ Primary antibodies including anti-MGMT (SPM287, santa cruz), anti-MSH2 (D24B5) anti-MSH6 (3E1) and anti-EGFR (#4267) from Cell Signaling were used in conjunction with appropriate HRP-conjugated secondary antibodies and chemiluminescence detection reagents (GE Healthcare) for visualization.

For immunohistochemistry analysis, paraffin sections were cleared and rehydrated through a series of Xylene then ethanol and water immersion. Following blocking, primary antibodies were incubated overnight at 4˚C, washed in PBS and incubated with secondary antibody for 1 h at room temperature. Immunohistochemical detection was performed using a Vectastain ABC kit (Vector Laboratories, Burlingame, CA) and diaminobenzidine (DAB; Merck, Darmstadt, Germany).

Allele specific PCR has been performed as previously described.^12^ Briefly, purified DNA was mixed with ddPCR Supermix for probes (186-3032, BioRad) along with custom FAM-HEX probes before droplet generation. A two-step PCR was run (95˚C for 10 min, then 45 cycles of 94˚C for 50 s, 59˚C for 60 s, followed by a final step at 72˚C for 10 min) before analysis on the QX200 Droplet Reader (BioRad).

### In Vivo Studies

All procedures involving animals were performed in accordance with the guidelines of the Canadian Council of Animal Care and the Animal Utilization Protocols, approved by the Institutional Animal Care Committee at the McGill.

NSG mice were orthotopically injected with various GSCs (20 000) and randomized into treatment and control groups. For radiation experiments, radiation was initiated 5 days after surgery and performed on the small animal X-RAD 225Cx (PXI, North Branford, CT, USA). A regime consisting of 20 Gy/10 fractions was delivered to treatment group and mice were monitored until ethical end points.

B57BL/6 mice were either subcutaneously injected with 1 million of cells or orthotopically injected at 20 000 using the murine glioma line GL261 or derivative lines and tumor growth monitored prior to treatment. For immunotherapy studies, treatment with anti-PD-1 (5 mg/kg, clone RMP1-14, Bio X Cell) was started 7 days following injection for 4 weeks or until ethical end-point was reached.

### Whole Genome Sequencing

Whole-genome sequencing was performed in Genome Quebec (McGill). Paired end libraries were prepared from GL261 lines and sequenced at low depth (40X) on the Illumina HiSeq 2000 platform. Raw sequencing reads were mapped to a mouse reference genome (mm10) and variants called using MuTect2. Raw variant sites were subjected to a series of quality filtering, such as the allelic and overall depth of coverage prior to further analysis.

### Extracellular Vesicle Studies

Cells were plated and conditioned media collected after at least 3 days of growth. Media was cleared of debris at 2000 g prior to concentration using Amicon Ultra-15 Centrifugal filter units (EMD Millipore, Billerica, MA) followed by centrifugation at 110 000 g for 120 mins. Vesicular pellets were washed once with PBS and spun down again at 110 000 g prior to utilization.

For pulldown enrichment studies, EVs were pre-cleared using unconjugated magnetic protein-A beads blocked with blocking solution (PBS, 5% Normal Goat Serum + 2% BSA) then incubated with rabbit anti-EGFR primary antibody (Cell Signalling #4267) and rocked overnight at 4˚C. Blocked protein-A beads (Thermofisher) were added the next day and rocked for 1hr at room temp. and then washed with blocking solution before downstream analysis.

## Results

### Identification of Hypermutant and Non-Hypermutant Recurrent GBM Subtypes

**Figure 1.**
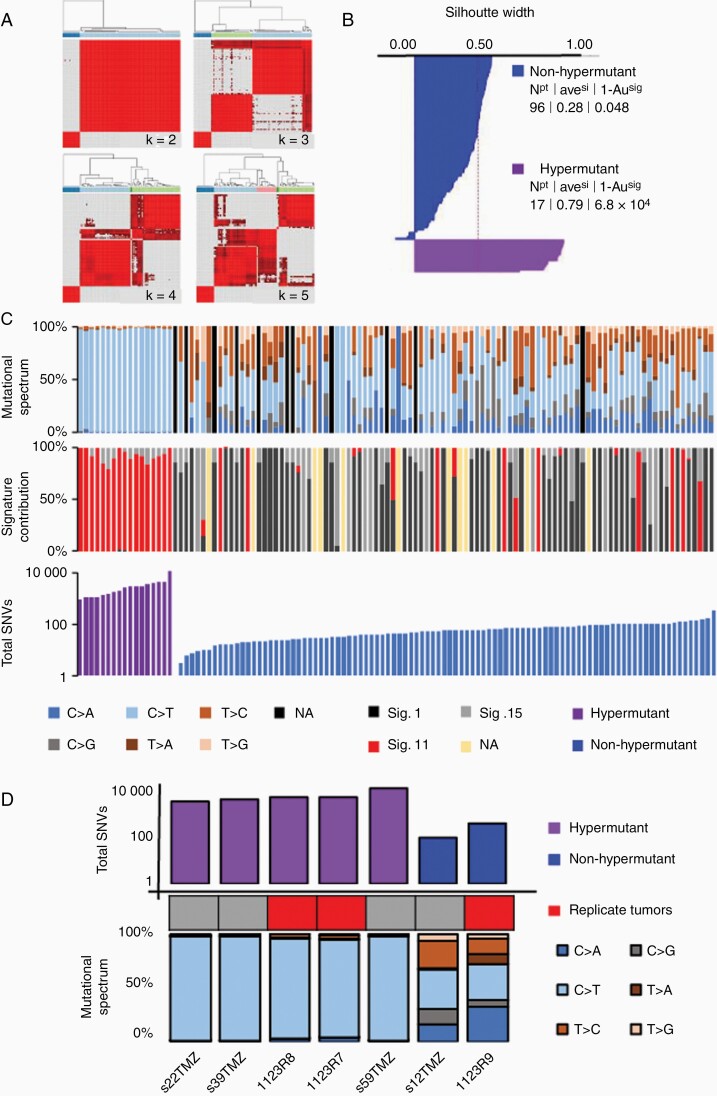
Identification of hypermutant and non-hypermutant subtypes and in vivo modelling of recurrence. (A) hierarchical clustering of 114 recurrent glioma exomes and (B) silhouette analysis of cluster stability. (C) Per-tumor analysis of mutational features including mutational spectrum (top) signature contribution (middle) and total single nucleotide variants (bottom) highlighting characteristics of hypermutant and non-hypermutant recurrent glioma (D) Exome analysis of in vivo models of GBM following treatment with TMZ demonstrating emergence of hypermutant and non-hypermutant subtypes.

In an attempt to characterize genetic subtypes which emerge following exposure to TMZ, we analyzed available exome datasets^7^ with matched primary and recurrent glioma (n = 114). We compared mutations at primary and recurrent stages to derive newly acquired mutations post-TMZ treatment, then carried out mutational signature analysis and performed consensus average linkage hierarchical clustering. Two clusters of greatest stability were derived which could classify post treatment glioma (Figure 1A, B) whilst increasing clusters to k = 3 or beyond resulted in greater misclassification of samples (Supplementary Figure 1). Cluster significance was evaluated using the R tool pvclust,^13^ which utilizes multiscale bootstrap resampling to calculate approximately unbiased *P*-values and both clusters were found to be statistically significant (Figure 1B). Following unbiased classification of these clusters, representative mutational signatures (COSMIC “catalogue of somatic mutations in cancer” signatures) were derived to show accumulation of C > T/G > A substitutions in cluster 2 (referred to hereafter as hypermutant), which was missing from cluster 1 tumors (referred to hereafter as non-hypermutant; Figure 1C). Characteristics of hypermutated tumors included *(i)* dramatic increase in mutational load across the genome compared to the matched primary tumor, *(ii)* acquired mutations in mismatch repair (MMR) genes including MSH2, MSH6 and PMS2, as well as *(iii)* enrichment of a transversion signature (COSMIC signature 11) at recurrence, indicating the effects of alkylating agents such as TMZ.^14^

The role of TMZ as a causal factor in driving hypermutation was corroborated using WES of patient derived xenografts (PDXs) (Figure 1D). TMZ treatment resulted in divergent outcomes, with the majority (5/7) of tumors emerging as hypermutant following TMZ whilst the minority (2/7) became non-hypermutant (Figure 1D). Importantly, analysis of exome sequencing from three replicate injections of a single parental glioma stem cell (GSC 1123) line we had previously published,^10^ we observed both hypermutated (1123R7, 1123R8) and non-hypermutated (1123R9) outcomes at recurrence following treatment with TMZ (Figure 1D).

### Hypermutant GSCs Exhibit MGMT-Independent TMZ Resistance and Radioresistance Compared to Non-Hypermutant GSCs

**Figure 2.**
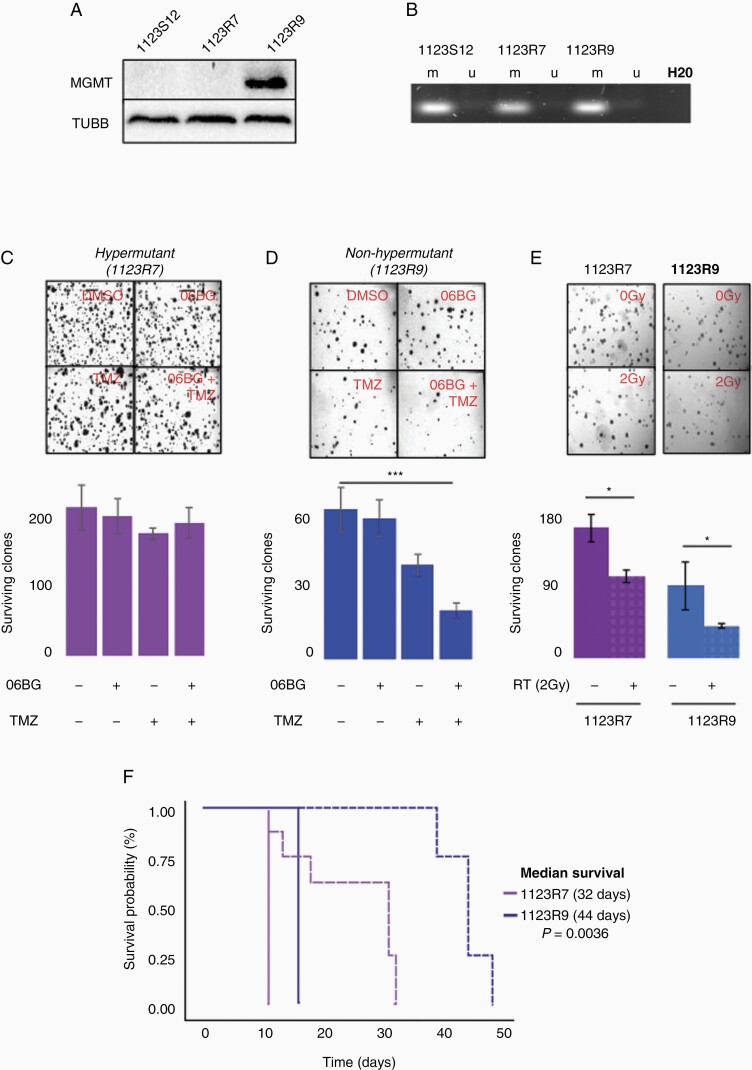
Response to further chemotherapy or radiotherapy differs between isogenic hypermutant and non-hypermutant GBM. (A) Immunoblot analysis of MGMT in isogenic hypermutant and non-hypermutant recurrent GBM and (B) assessment of MGMT methylation status using methylation-specific polymerase chain reaction analysis showing maintenance of MGMT methylation despite upregulation in 1123R9. Assessment of clonogenic survival in (C) hypermutant and (D) non-hypermutant tumors against combinations of TMZ and/or 06BG (*N* = 3). (E) Assessment of in vitro clonogenic survival against a single dose of 2Gy in hypermutant and non-hypermutant tumors. (F) *in vivo* assessment of tumor response to a fractionated radiotherapy regime (20Gy/10 fractions) in hypermutant and non-hypermutant GBM.

We and others have previously demonstrated that O6-methylguanine DNA methyltransferase (MGMT) is upregulated in a subset of glioma after TMZ where MGMT mediates the primary mode for chemoresistance.^10^ Immunoblot confirmed MGMT upregulation only in non-hypermutant 1123R9 GSCs suggesting its role in protection from mutational acquisition (Figure 2A).^10^ Methylation specific PCR revealed maintenance of MGMT methylation in both MGMT-negative 1123R7 and MGMT-expressing 1123R9 GSCs (Figure 2B) indicating an alternate method of upregulation similar to prior reports.^15^ As expected, depletion of MGMT in hypermutant 1123R7 GSCs revealed no change in clonogenic survival when challenged with TMZ (Figure 2C). In comparison, 1123R9 GSCs treated with TMZ and 06-BG showed decreased clonogenic survival compared to control (*P* < .0005; Figure 2D). We also tested several other chemotherapeutic agents and found that hypermutant 1123R7 GSCs demonstrated an increase in resistance to Cisplatin compared to non-hypermutant 1123R9 GSCs (Supplementary Figure 2A–E).

We then assessed whether these cells differ in their response to RT. Clonogenic assessment of survival revealed that hypermutant 1123R7 GSCs were less responsive to RT compared to non-hypermutant 1123R9 GSCs (SF2 = 0.67 vs 0.48, *P* = .032; Figure 2E). This differential sensitivity to RT was confirmed through an in vivo study as hypermutant 1123R7 tumors exhibit less response to a fractionated RT regime (20 Gy/10 fractions) compared to non-hypermutant 1123R9 (median survival of 32 days vs 44 days respectively; *P* = .00318; Figure 2F). This suggests that hypermutation at recurrence may indicate a limited response to re-irradiation although the mechanisms by which this occurs are still to be elucidated.

### Hypermutation Predicts Immunogenicity in Immunocompetent Models

Tumor mutational burden predicts immunotherapy response across multiple cancer types.^16^ To investigate whether this was true for GBM, we generated recurrent GBM by orthotopically injecting the mouse glioma line GL261 into NSG mice prior to treatment with either TMZ (120 mg/kg) or vehicle as previously published.^10^ From these experiments, we derived three lines (1) a primary untreated line (GL261-ctr), (2) a recurrent line following TMZ-treatment (GL261-TMZ), and (3) an additional recurrent line (GL261-TMZ+06BG) which was similarly isolated from a TMZ-treated recurrent tumor then treated for an additional 4 weeks in vitro with TMZ (50 μM) and O6BG (25 μM). Of note, these lines were generated in an immune compromised NSG mouse to overcome negative selection by the immune system, which may result in suppression of hypermutated phenotype.

**Figure 3.**
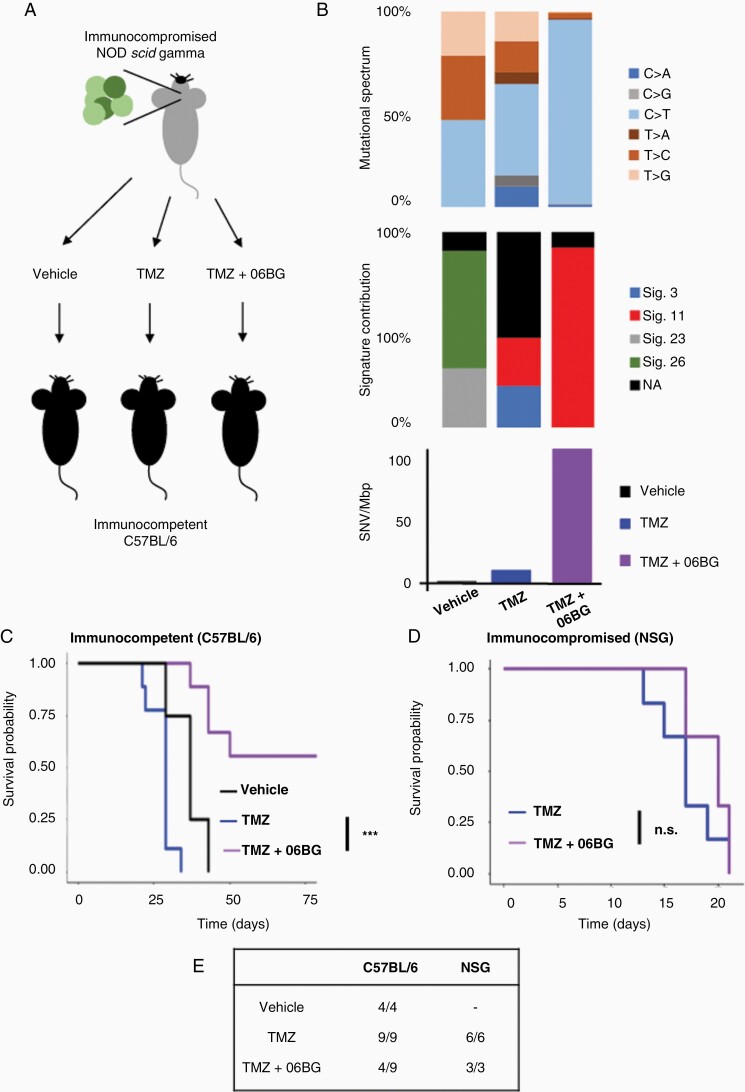
Hypermutation at recurrence predicts immunogenicity in subcutaneous settings. (A) Generation of isogenic recurrent GBM using orthotopic injection of GL261 into immunocompromised NSG mice and TMZ treatment followed by assessment of phenotype in immune competent C57BL/6 mice. (B) Exome analysis of GL261 lines following vehicle, TMZ or TMZ+06BG treatment. Survival outcome following subcutaneous injection of Gl261 lines into (C) immune competent C57BL/6 mice or (D) immunocompromised NSG mice. (E) Summary table of survival highlighting the immune rejection of over half (5/9) of hypermutant GL261 in immune competent C57BL/6 mice.

We then investigated the mutational landscape of primary (GL261-ctr) and recurrent GL261 lines (GL261-TMZ, GL261-TMZ+O6BG) by performing whole genome sequencing (Figure 3B). As expected, the greatest increase in mutational burden was seen in GL261-TMZ+O6BG cells. These cells displayed all the features characteristic of hypermutant tumors which included high mutational burden (~112 SNV/Mbp) and enrichment for TMZ-associated mutational signature 11 (~90%; Figure 3B). In contrast, GL261-TMZ cells gained fewer mutations (~10 SNV/Mbp) consistent with the limited TMZ impact and only a minor enrichment (~24%) for mutational signature 11 (Figure 3B). As expected, GL261-ctr cells were the most similar to the un-injected parental GL261 cell line and had an absence of TMZ specific mutational signature 11.

We next sought to evaluate whether hypermutation was predictive of greater immunogenicity. To this end, we subcutaneously injected GL261 lines into immune competent C57BL/6 mice and evaluated growth (Figure 3C). We found a striking difference in tumor growth in C57BL/6 mice, with significant growth delay of the hypermutant GL261-TMZ+O6BG in C57BL/6 compared to GL261-TMZ (*P* = 6.42 × 10^-13^) or GL261-ctr (*P* = 2.31 × 10^-8^). Notably, over half (5/9) of C57BL/6 mice injected with hypermutated GL261-TMZ+O6BG displayed rejection of allograft formation (Figure 3C, Supplementary Figure 3A). We also performed injections into immunocompromised NSG mice to infer the role of the immune system in preventing tumor formation from hypermutant GL261 cells (Figure 3D). Both GL261-TMZ and GL261-TMZ+O6BG rapidly grew tumors following subcutaneous injection, giving evidence that the failure of tumor formation observed in C57BL/6 mice after injection of hypermutant GL261-TMZ+O6BG cells is likely due to an immune rejection (Figure 3E). Together, these results suggest that injection of subcutaneous hypermutant tumors are sufficient to elicit an immune response in C57BL/6 mice.

### Hypermutant Recurrent GBM Exhibits Limited Immunogenicity in Orthotopic Settings

**Figure 4.**
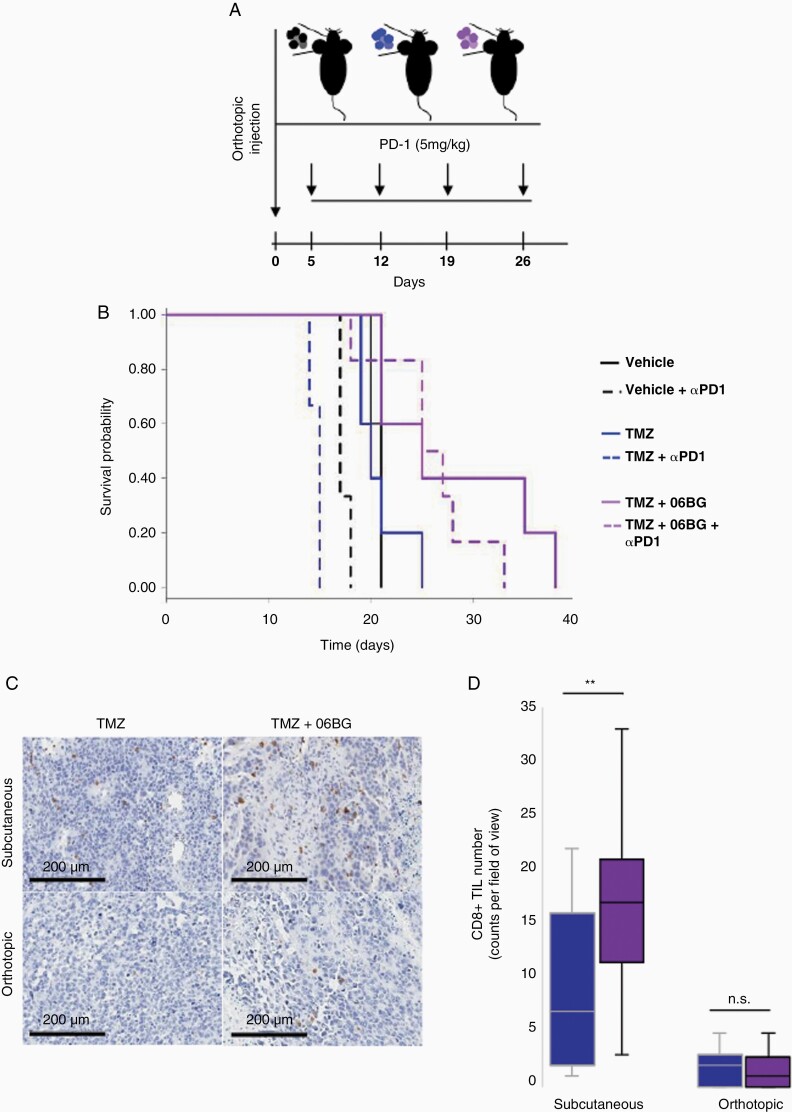
Hypermutation is not sufficient to induce immunogenic response in orthotopic settings. (A) Outline of anti PD-1 therapy regime consisting of weekly dose of therapy to mice which have had orthotopic injection of hypermutant or non-hypermutant GL261. (B) Survival outcome in mice following treatment with anti-PD-1 therapy. (C) Immunohistochemical evaluation of CD8 in GL261 subcutaneous and orthotopic xenografts. (D) Quantification of CD8+ cell infiltration in GL261 subcutaneous and orthotopic xenografts.

Given our observation of robust immune response to subcutaneous hypermutant xenografts, we investigated whether this can be recapitulated in an orthotopic microenvironment and whether hypermutation might be predictive of anti-PD-1 immune checkpoint inhibitor response. Following orthotopic implantation of GL261 cells into immunocompetent C57BL/6 mice, we treated with mAb PD-1 over 4 weeks (Figure 4A). In contrast to the robust immune response leading to xenograft rejection in over half of GL261-TMZ+O6BG injected into the flank of C57BL/6 mice, orthotopic injection of hypermutant GL261 cells resulted in 100% of brain tumor formation in GL261-TMZ+O6BG mice. Similarly, GL261-ctr and GL261-TMZ also engrafted with 100% success rate (Figure 4B). In the absence of PD-1 inhibitor, there was no statistical difference in survival for mice with hypermutated GL261-TMZ+O6BG tumors compared to GL261-ctr (21 days vs 25 days; *P* = .072) or GL261-TMZ (20 days vs 25 days; *P* = .091). Comparison of anti-PD1 treated mice resulted in a survival advantage for hypermutant GL261-TMZ+O6BG mice compared to GL261-crt (26 days vs 15 days; *P* = 2.16 × 10^-4^) as well as compared to non-hypermutant GL261-TMZ mice (26 days vs 17 days; *P* = 1.54 × 10^-3^). Interestingly, we found no survival improvement with use of anti-PD1 treatment on hypermutant GL261 tumors consistent with limited efficacy of ICI monotherapies in GBM.^17–22^

Given the striking difference in immune response between orthotopic vs subcutaneous tumors, we then investigated whether this could be due to differences in immune cell infiltration. Consistent with this hypothesis, CD8 T-cell infiltration was significantly greater in subcutaneous hypermutant GL261 tumors compared to non-hypermutant tumors (*P* = .0038; Figure 4C, D; Supplementary Figure 4A). In contrast, orthotopic tumors had limited CD8 T-cell infiltration and this was observed in both non-hypermutant GL261-ctl as well as hypermutant GL261-TMZ+O6BG tumors (*P* = .63). Analysis of T-regulatory cells (FOXP3) revealed that there was robust presence of positive cells in subcutaneous tumors but an absence of FOXP3 positive cells in orthotopic tumors (Supplementary Figure 4B). In contrast, macrophages (F4/80) were present in both subcutaneous and orthotopic tumors, however we did not observe any differences between hypermutant and non-hypermutant tumors (Supplementary Figure 4B). No differences were observed in CD14 staining between hypermutant and non-hypermutant tumors (Supplementary Figure 4B).

### Quantification of Hypermutant Mutational Burden Using a Minimal Gene Panel

**Figure 5.**
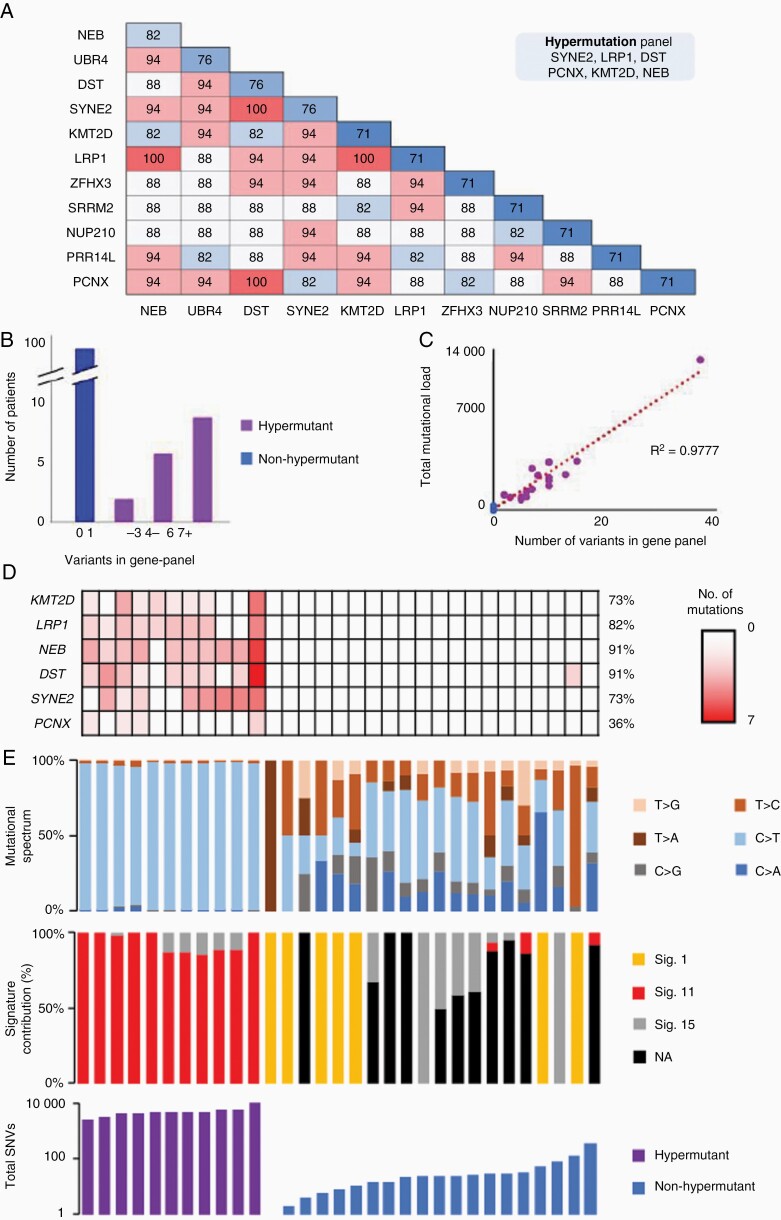
Identification of a targeted gene panel to diagnose hypermutation in recurrent GBM. (A) The frequency of identification of hypermutant tumors in the discovery set using combinations of 2-genes. (B) Specificity using 6-gene signature of LRP1, NEB, PCNX, KMT2D, DST and SYNE2 to discriminate between hypermutant and non-hypermutant tumors. (C) Correlation of mutational load across the 6-gene signature to total mutational burden across the whole exome. (D) Identification of hypermutant tumors in an independent validation set using the 6-gene panel. (E) Tumor specific characteristics including mutational spectrum (top panel), signature contribution (middle panel) and total mutational burden (bottom panel).

Despite the loss of MMR functionality in hypermutant glioma, microsatellite instability is similar to that of non-hypermutant tumors^5^ and is a poor diagnostic marker for hypermutation. Similarly, MMR gene mutations whilst now accepted to be causal in driving hypermutation are not ubiquitously mutated in all hypermutant tumors^23,24^ nor do they indicate the level of mutational burden, creating a need for alternative approaches to identify and quantify hypermutation. To derive a minimal gene panel which could diagnose hypermutation, we first analyzed paired primary and recurrent GBM sequencing datasets (taken from ref 12; n = 114 patients). Mutations were spread over the whole genome, with no single chromosome exclusively hypermutated in hypermutant GBM (Supplementary Figure 5A). With respect to gene-level mutational accumulation, we observed that while many genes were exclusively mutated in hypermutant tumors, no individual gene was consistently mutated in all hypermutant GBM, indicating that a combination of genes would be necessary for accurate discrimination between hypermutant and non-hypermutant tumors (Supplementary Figure 5B). Strikingly, among genes exclusively mutated at recurrence in hypermutant GBM, a combination of six genes (denoted as *Hyper-6*: LRP1A, PCNX1, KMT2D, DST, SYNE2 and NEB) was able to successfully identify 100% of hypermutated tumors (Figure 5A). Non-hypermutated tumors were not observed to gain mutations in any of these six genes at recurrence, allowing 100% accuracy of diagnosis when used upon the discovery dataset (Figure 5B). Comparison against random gene set permutations highlighted the accuracy of the *Hyper-6* panel, as the maximum accuracy reached with these was 52%, well below the 100% accuracy achieved when using the *Hyper-6* (Supplementary Figure 5C). Further investigation of our panel revealed that mutational burden was highly correlated with the number of mutations over these six gene sequences but not random gene sets (R^2^ = 0.9777l; Figure 5C, Supplementary Figure 5D).

Validation of the *Hyper-6* panel was performed upon an independent dataset comprised of publicly available exome sequencing experiments from 31 glioma.^6,25–27^ Analysis of these 31 tumors using the *Hyper-6* panel identified 12 tumors as hypermutant (Figure 5D). Eleven of those tumors were found to be hypermutant upon examination of their exome sequencing data (Figure 5E). In all cases, non-hypermutant tumors did not gain any mutation in the *Hyper-6* panel of genes. Genomic mutational burden was again highly correlated with the number of mutations over these 6 genes (R^2^ = 0.8922; Supplementary Figure 5E). Notably, the GL261 hypermutant tumors also showed a gain in mutations in several of the hypermutant genes which paralleled mutational burden (R^2^ = 0.9877; Supplementary Figure 5F)

### Extracellular Vesicles Harbor DNA with a Hypermutation Signature

**Figure 6.**
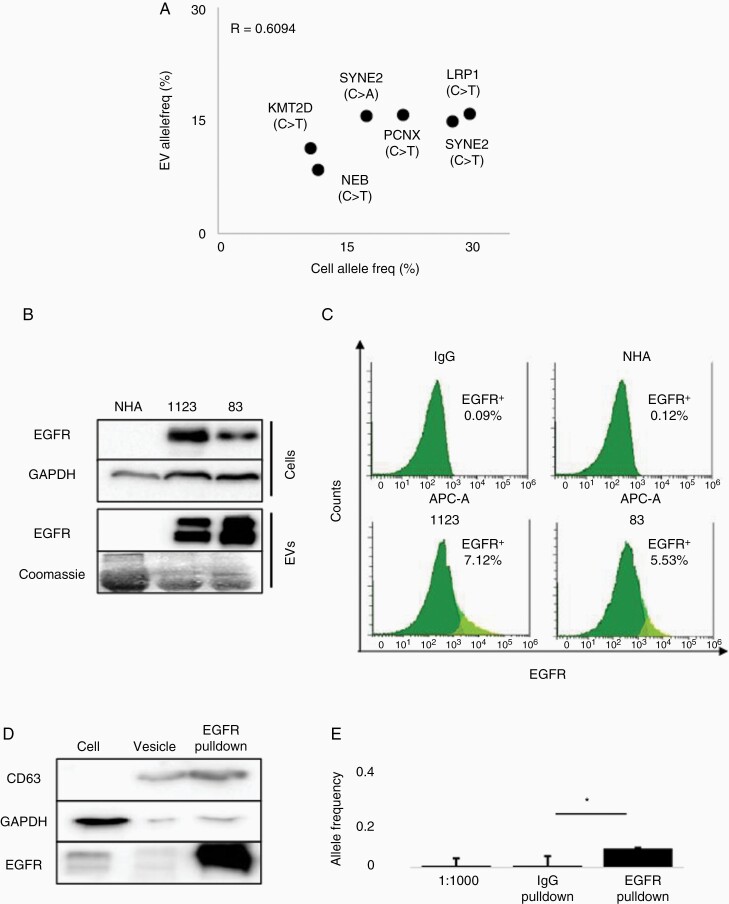
Detection of 6-gene signature in extracellular vesicles. (A) Correlation graph of allele frequency of mutations from cells and vesicles as detected by targeted sequencing. (B) Immunoblot analysis comparing EGFR expression from cells and vesicles. (C) Nano-flow cytometry comparison of vesicles from normal human astrocytes (NHA) to GBM cell vesicles (*N* = 3). (D) Immunoblot analysis of immunoprecipitated from EGFR pulldown of vesicles showing loss of cell marker GAPDH and gain in vesicle marker CD63. (E) Digital droplet PCR based detection of LRP1 mutation comparing IgG and EGFR vesicle enrichment methods mixed vesicle solutions from hypermutant cell and NHA (*N* = 2).

Extracellular Vesicles (EVs) are acellular structures released from cells which carry DNA, RNA and proteomic cargo representative of the parental cell.^28–30^ To determine whether the hypermutant DNA was encapsulated in the vesicular compartment, we isolated EVs from hypermutant 1123R7 GSCs and performed targeted sequencing for the 6-gene signature previously identified. Comparison of variants from hypermutant 1123R7 GSCs and 1123R7 EVs revealed that all six mutations could be detected using either source of DNA (Figure 6A). The variant allele frequency (AF) of the six variants was highly correlated between the GSC tumor DNA and EV DNA (R^2^ = 0.6094; Figure 6A), suggesting that vesicular DNA may closely represent the genomic landscape of hypermutant GBM cells. We further investigated this correlation using digital droplet PCR (ddPCR) for LRP1 mutation found in 1123R7 hypermutant GSCs, observing a closely related variant AF between these two sources of DNA (AF = 0.23 vs 0.19, Supplementary Figure 6A, B).

As EVs harbor proteins present in originating cells, we then sought to determine whether cancer-specific EVs can be discriminated based upon expression of oncogenic proteins associated with specific disease states.^28,31^ This is especially important for future translation towards liquid biopsy use, as tumor EVs in biofluids are mixed with EVs secreted by non-tumor cells, leading to loss of sensitivity of targeted sequencing methods.^32^ Epidermal growth factor receptor (EGFR) is a transmembrane protein overexpressed in over half of GBM and sorted into EVs harboring *EGFR* gene amplification, which reflects EGFR oncogenic alterations from the original GBM tumor, including TMZ-resistant GSCs.^10,28,31,33^ Protein analysis of GSCs 1123 and 83 identified overexpression of EGFR in these lines compared to normal human astrocytes (NHAs), which expressed minimal EGFR (Figure 6B). Analysis of EVs derived from these cells showed a similar pattern where EGFR was present only in EVs from the GSCs, but not in NHA EVs (Figure 6B). Nano-flow cytometry confirmed the differential EGFR expression between NHA and GSCs, suggesting the existence of a robust subset of EGFR-positive EVs released from the GSCs cells (Figure 6C). EGFR immunoprecipitation (IP) of EVs isolated from 1123R7 GSCs, which was readily able to enrich for a CD63 vesicular fraction (Figure 6D) confirmed our findings. Thus, EGFR pulldown methods provide the means to discern tumor-EVs from EVs secreted by non-tumor cells.

To explore whether IP-based enrichment can increase sensitivity of detection, EVs isolated from NHAs or 1123 GSCs were mixed at a ratio of 1:1000 and then analyzed for AF of hypermutant DNA using ddPCR for LRP1 mutant and wild-type sequences. Vesicles isolated from 1123R7 hypermutant GSCs closely mirrored cells, demonstrating a variant AF of 43.867%. From the unenriched and IgG pulldowns, we observed an AF of 0.351% and 0.120%, respectively (Figure 6E). In comparison, AF of hypermutant LRP1 variant in vesicles following EGFR pulldown was found to be 6.504%, representing a 21.6-fold increase, improving sensitivity compared to unenriched pulldown (*P* = .018; Figure 6E). Together our findings suggest the utility of using tumor enriched markers to pulldown tumor specific EVs which contain genomic material which may be useful in the development of minimally invasive diagnostic methods for specific disease states such as hypermutation.

## Discussion

Despite decades of investigation, GBM recurrence remains inevitable despite aggressive upfront treatment with combined resection and chemoradiation. Immunotherapy has emerged as a core therapeutic option in multiple cancer types over the past few years, enabling the reactivation of immune responses against neo-antigen rich tumor cells and improved outcome for patients. Highlighting the success of this treatment approach across diseases, the FDA has now approved specific ICIs for use in deficient MMR/MSI-H tumors irrespective of cancer type.^34^ Immunotherapy in brain tumors has failed to meet the primary endpoint using ICIs in several clinical trials for both primary and recurrent GBM.^35,36^ Our work supports the emergence of hypermutation post-TMZ resistance as a distinct subtype from non-hypermutant recurrent GBM. We propose that hypermutant recurrent GBM are phenotypically and immunologically distinct from their counterparts, able to be recognized by immune cells and evoke a greater immune response. However, this response is only observed in subcutaneous contexts, as immune rejection of hypermutant cells is not observed in orthotopic contexts and immune response cannot be rescued by anti-PD-1 ICI therapy. Finally, we show that genomic material from hypermutant tumors is present in acellular vesicles which may be useful for the development of minimally invasive diagnostic tests.

One reason for the failure of immunotherapy in GBM is the reportedly “cold” immunological microenvironment. Compared to immunologically “hot” tumors which present with high levels of cytotoxic T-cell infiltration within the tumor mass, “cold” tumors such as GBM have limited infiltration and is often cited as the reason for limited response to immunotherapies.^37,38^ Increasing evidence suggests that immune trafficking to the brain is not as limited as previously reported.^39^ Active suppression of immune infiltration by local microenvironmental factors might contribute to an immune “cold” system for GBM.^37^ Acquisition of hypermutation during TMZ treatment increases the mutational burden across the genome (reviewed by our group in^40^) and has been suggested as one way in which a “cold” tumor can be converted into a “hot” tumor.^41^ Our observations suggest that increasing mutational burden is not sufficient *per se* to convert GBM to a “hot” tumor. Furthermore, our results highlight that the brain microenvironment suppresses immune response allowing even highly immunologically targetable tumors such as hypermutant GBM to grow minimally impeded by immune responses. Cells specific to the central nervous system such as microglia have been shown to be able to contribute to active immune suppression via release of molecules such as FASL and B7-H1 which drive apoptosis of T-cells.^42,43^ Further research to identify the mechanisms driving poor response of our PDX models in orthotopic contexts will provide new insights into specific therapies to modulate immune response for CNS tumors.

Several clinical trials using ICIs in primary and recurrent GBM setting without biomarkers selection (high-MSI or TMB) have failed to show a survival benefit compared to standard of care. These studies include Checkmate-498 (NCT02617589) testing Nivolumab in MGMT unmethylated GBM, and Checkmate-548 testing Nivolumab in MGMT methylated GBM, in combination with standard chemoradiation therapy.^35^ While using hypermutation as a biomarker for ICI therapy has gained increased interest, standardized definition of this state of hypermutation has yet to be established. In recurrent GBM settings, one trial testing Pembrolizumab (NCT02658279) is underway using patient selection according to hypermutant status; tumors as either having a mutation count of >30 using the MSK-IMPACT panel, mutation in one or more MMR genes (MLH1, MSH2, MSH6, PMS2, POLE or POLD), or high MSI. Yet this definition of an immunologically susceptible hypermutated tumor is extrapolated from other diseases immunologically and genetically distinct from GBM,^16^ which might likely limit its accuracy in recurrent GBM. The simple six-gene panel identified in our study represents genes exclusively mutated in TMZ-driven hypermutant recurrent GBM and highly correlated with TMB.

Whilst we previously observed that there was a high number of vesicles release in vitro release from 1123R7 cells,^10^ whether this is maintained in vivo was not evaluated. Furthermore, whilst we have provided an initial description of pulldown methods for enriching tumor specific EV-DNA based on differential protein enrichment of EGFR on tumor vs non-tumor EVs, the relevance for these methods for liquid biopsy detection of hypermutation are still not established for GBM. Microfluidic devices which use antibody based enrichment methods to increase detection thresholds are gaining interest and preliminary evidence from these reports suggest the validity of this approach.^44^

Treatment for recurrent GBM patients has stagnated for over a decade and most patients receive lomustine, Avastin, or re-irradiation as standard protocol regardless of molecular features. We propose that hypermutant status defines a unique subgroup of recurrent GBM which may require a different approach to therapy compared to non-hypermutant GBM. This study lays the groundwork to further evaluate how hypermutation can be integrated into personalized treatment to ultimately improve the dismal outcome of patients with recurrent GBM.

## Supplementary Material

vdac076_suppl_Supplementary_Data

## Notes

**Authorship statement:** Experimental contributions (P.D., B.M., D.C., K.G., A.P.). Conceptualization and manuscript preparation (P.D., B.M., S.S., B.A., J.R.). Manuscript review (P.D., B.M., S.S., F.J., J.N.S., G.K., Y.R., J.R., B.A.).
