# Supplementary material for: Detection of temozolomide-induced hypermutation and response to PD-1 checkpoint inhibitor in recurrent glioblastoma
Source: Neurooncol Adv. 2022 May 23;4(1):vdac076. doi: 10.1093/noajnl/vdac076 (PMC9252128; doi:10.1093/noajnl/vdac076)

**Supplementary Table and Figures**

**Supplementary figure 1** (A) Silhouette plot for n=3 clusters showing misclassification of cluster 1.


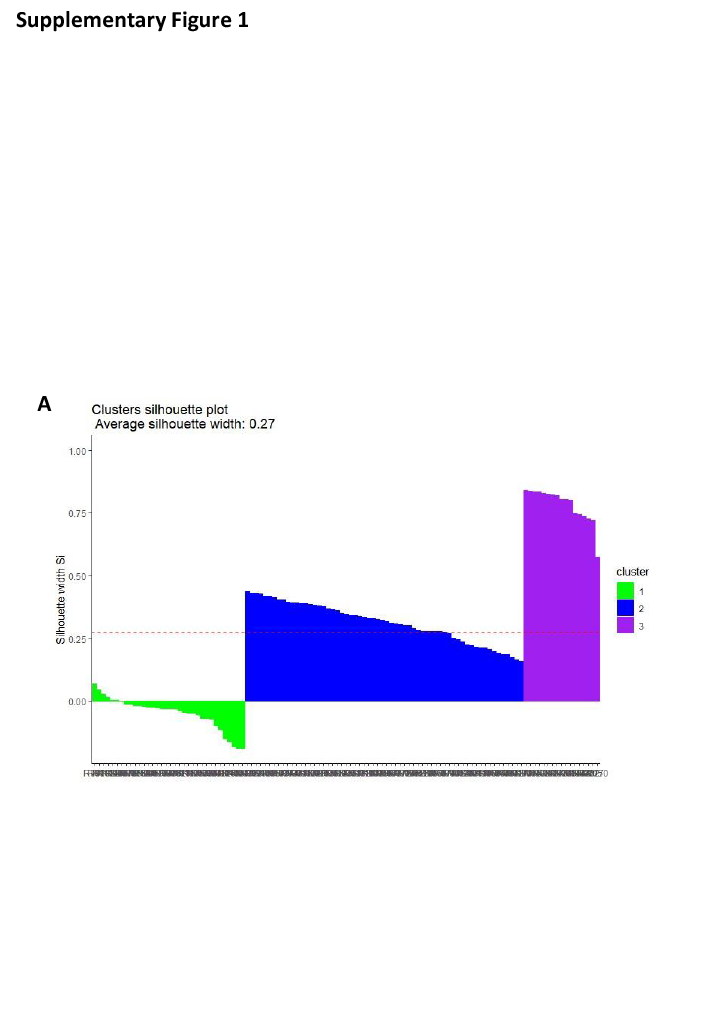


**Supplementary figure 2** (A). Effect of temozolomide on primary (1123S12), hypermutant (1123R7) and non-hypermutant (1123R9) recurrent GBM cells. Comparison of effect of (B) Irinotican, (C) Doxurubicin, (D) Cisplatin and (E) Vincristine on hypermutant and non-hypermutant cells in vitro


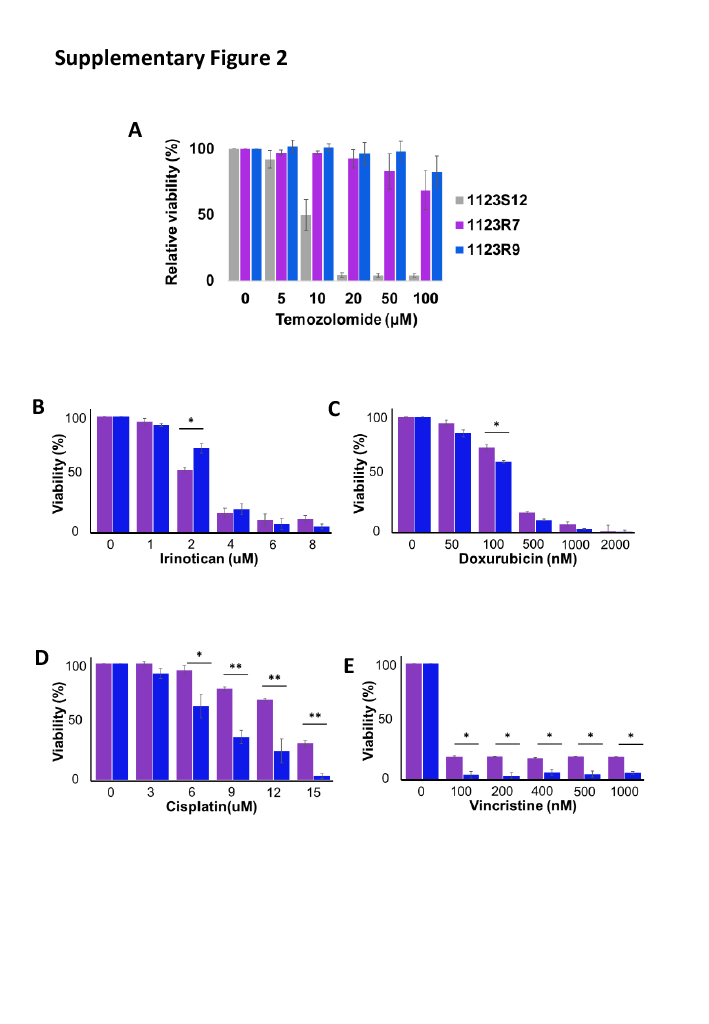


**Supplementary figure 3** (A). Subcutaneous tumor growth for parental GL261-vehicle, non-hypermutant GL261-TMZ and hypermutant GL261-TMZ+O6BG grown in C57BL/6 mice


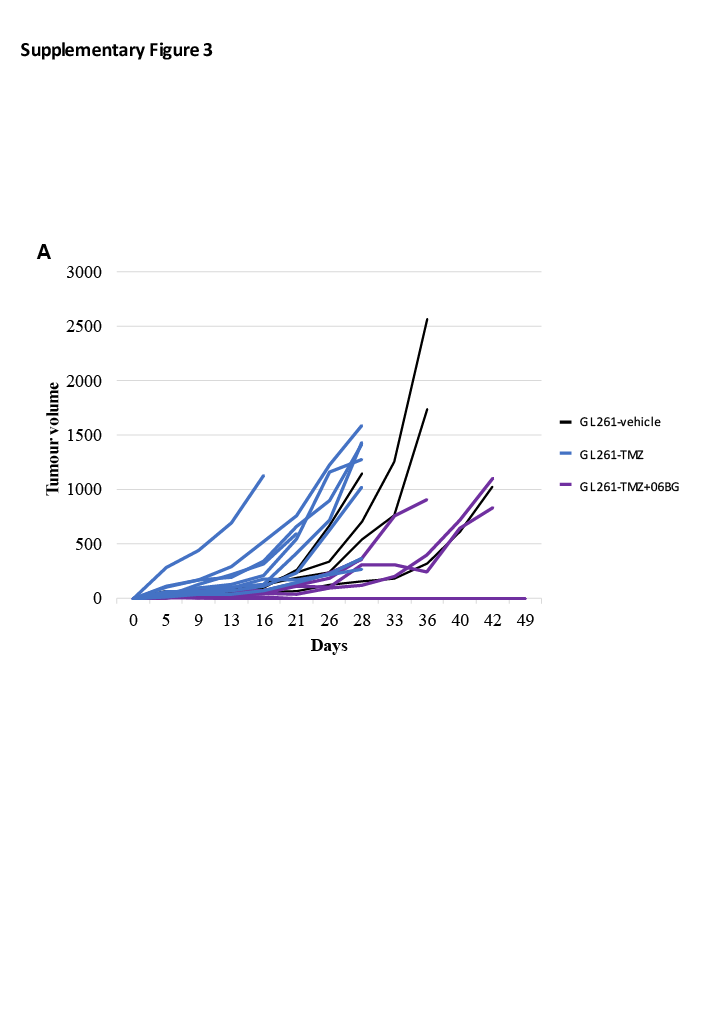


**Supplementary figure 4** (A) IHC for CD8 cells (brown) in non-hypermutant (Vehicle) and hypermutant (TMZ + 06BG) GL261 tumors in subcutaneous and orthotopic settings. (B) IHC for D3(brown)/F480(pink), FOXP3(pink) and CD14 (brown) in non-hypermutant (Vehicle) and hypermutant (TMZ + 06BG) GL261 tumors in subcutaneous and orthotopic settings.


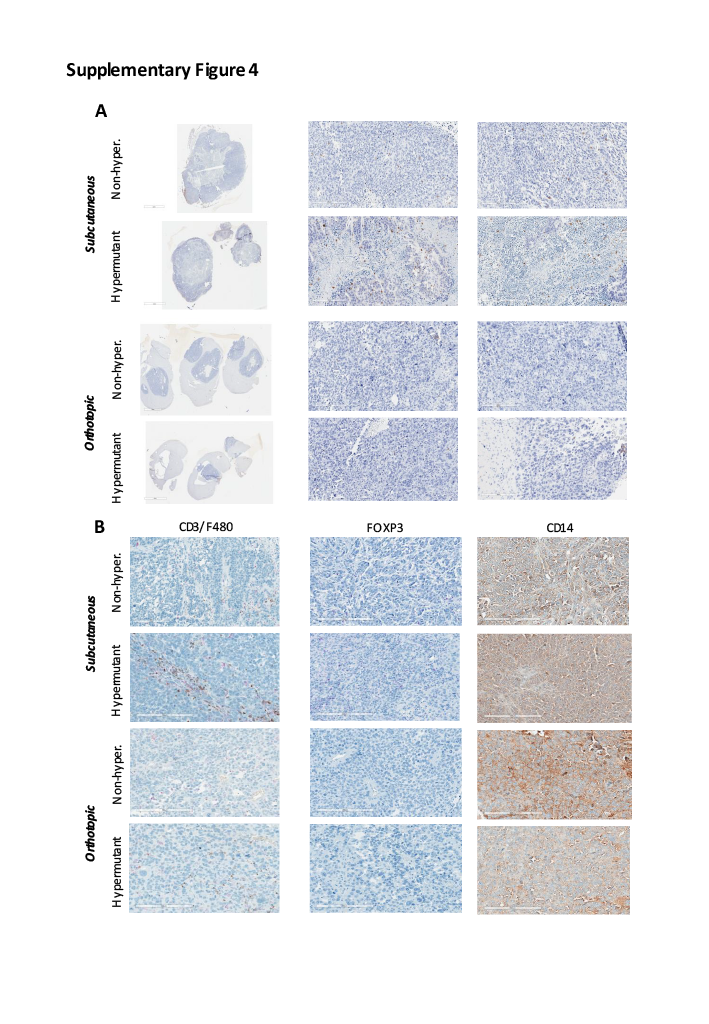


**Supplementary figure 5** (A). Mutational rate across the genome between hypermutant and non-hypermutant specimens. (B) Comparison of gene propensity to be enriched exclusively in hypermutant tumors (C) Random gene set permutation capacity to predict hypermutant state accurately (D) Correlation of random gene sets to mutational burden compared to the Hyper-6panel. (E) Validation dataset correlation plot of Hyper-6 mutations and mutational burden across the genome. (F) Mutational burden in GL261 derived lines and hyerp-6 panel genes


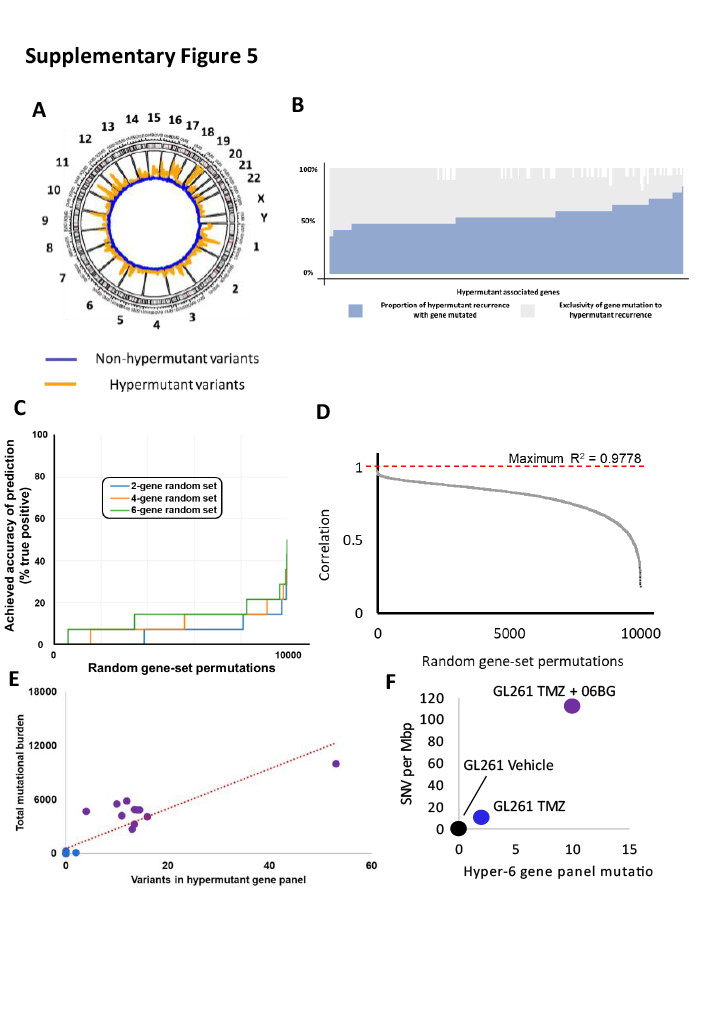


**Supplementary figure 6** (A). ddPCR plot for LRP1 in cells and vesicles isolated from conditioned media. (B) Comparison of allele frequency of LRP1 in cell and vesicle fractions


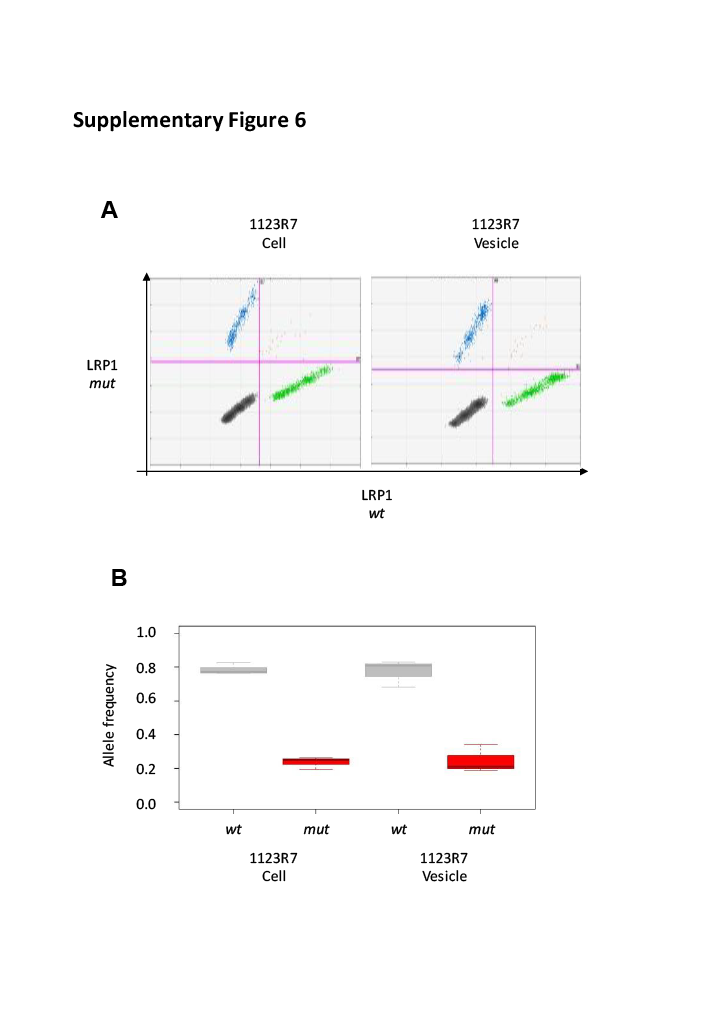

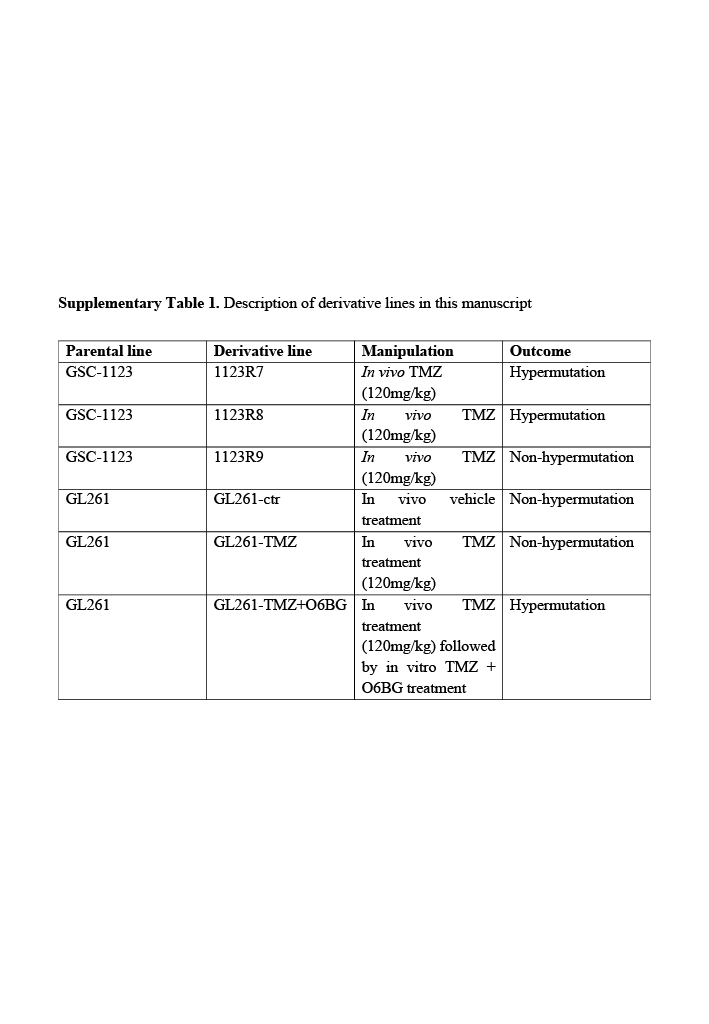

Supplement: vdac076_suppl_Supplementary_Data [file vdac076_suppl_supplementary_data.docx]
